# Indomethacin: The Interplay between Structural Relaxation, Viscous Flow and Crystal Growth

**DOI:** 10.3390/molecules27175668

**Published:** 2022-09-02

**Authors:** Roman Svoboda, Daniela Košťálová, Miloš Krbal, Alena Komersová

**Affiliations:** 1 Department of Physical Chemistry, Faculty of Chemical Technology, University of Pardubice, Studentská 573, 532 10 Pardubice, Czech Republic; 2 Center of Materials and Nanotechnologies (CEMNAT), Faculty of Chemical Technology, University of Pardubice, nam. Cs legii 565, 530 02 Pardubice, Czech Republic

**Keywords:** amorphous indomethacin, DSC, crystal growth, structural relaxation, viscous flow, particle size

## Abstract

Non-isothermal differential scanning calorimetry (DSC) was used to study the influences of particle size (d_aver_) and heating rate (q^+^) on the structural relaxation, crystal growth and decomposition kinetics of amorphous indomethacin. The structural relaxation and decomposition processes exhibited d_aver_-independent kinetics, with the q^+^ dependences based on the apparent activation energies of 342 and 106 kJ·mol^−1^, respectively. The DSC-measured crystal growth kinetics played a dominant role in the nucleation throughout the total macroscopic amorphous-to-crystalline transformation: the change from the zero-order to the autocatalytic mechanism with increasing q^+^, the significant alteration of kinetics, with the storage below the glass transition temperature, and the accelerated crystallization due to mechanically induced defects. Whereas slow q^+^ led to the formation of the thermodynamically stable γ polymorph, fast q^+^ produced a significant amount of the metastable α polymorph. Mutual correlations between the macroscopic and microscopic crystal growth processes, and between the viscous flow and structural relaxation motions, were discussed based on the values of the corresponding activation energies. Notably, this approach helped us to distinguish between particular crystal growth modes in the case of the powdered indomethacin materials. Ediger’s decoupling parameter was used to quantify the relationship between the viscosity and crystal growth. The link between the cooperativity of structural domains, parameters of the Tool-Narayanaswamy-Moynihan relaxation model and microscopic crystal growth was proposed.

## 1. Introduction

Indomethacin (IMC) is a non-steroidal anti-inflammatory drug (NSAID), which blocks the synthesis of prostaglandins by inhibiting cyclo-oxygenase (that converts arachidonic acid into cyclic endoperoxides). The antipyretic and analgesic therapeutic effects of IMC are commonly utilized to suppress inflammation, fever and pain. In particular, IMC is used to treat inflammation in rheumatoid arthritis by reducing swelling and tenderness. A similar principle is also applied in the IMC-based treatment of periarthritis, osteoarthritis, spondylosis deformans and acute gout. Indomethacin is considered, compared to the other NSAIDs, to be a more potent vasoconstrictor, consistently decreasing the cerebral blood flow and inhibiting CO_2_ reactivity. The significant acute and long-term toxicity of IMC are the reasons for a precise dosage being crucial in IMC-based treatments [1,2,3,4,5].

The low water solubility of IMC (<1 mg·L^−1^ at 25 °C [6]) impairs its clinical effectivity. Therefore, the possibility of using IMC in the amorphous form is under serious consideration, as this would greatly enhance the bioavailability of IMC due to the significant augmentation of the drug’s solubility [7]. The higher reactivity and lower cohesion of the amorphous phase (compared to the crystalline one) is, however, also a reason for its generally lower thermal stability. Amorphous IMC belongs to the III GFA (glass-forming ability) class [8,9], which means that, during the specified repeated heating/cooling cycle in the differential scanning calorimeter (DSC), the material remains amorphous. The standardized cyclic temperature program [8] is performed as follows: (1) the heating of the material above melting temperature T_m_ at 10 °C·min^−1^; (2) the cooling of the melt below the glass transition temperature T_g_ at 20 °C·min^−1^; (3) and the heating of the formed glass above T_m_ at 10 °C·min^−1^. Nonetheless, in practice, the aging, powdering or even slight surface damage to the amorphous IMC results in its transformation to the crystalline form. Such spontaneous/uncontrolled devitrification leads to the unexpected decrease in the IMC bioavailability, which can have life-threatening consequences for the patients. This creates the increased technological demand for a detailed description of the IMC amorphous-to-crystalline transformation. Considering that IMC is also often used as a model drug, surprisingly little information can be found regarding the overall thermo-kinetic characterization of the key physicochemical processes associated with amorphous IMC. This may be the consequence of IMC exhibiting interesting microscopic crystal growth properties (which seem to be the dominant focus of the research dealing with the thermal properties of IMC): the violation of the Stokes-Einstein law [10] at low temperatures [11] (which is relatively common for fragile glass formers [12]), and the so-called diffusion-less glass-crystal (GC) growth [13]. In addition to the above-described macro/microscopic crystal growth, the structural relaxation (glass transition kinetics) and thermally induced decomposition are also among these key thermo-kinetic properties. Whereas the decomposition kinetics are usually studied purely from a technological point of view to determine the temperature limitations for the drugs in processing, the structural relaxation is closely interlinked with the viscous flow, and both these types of structural rearrangements then directly influence the crystal growth (both below and above T_g_) [10,11,12,13].

The aim of the present paper is to provide a complete thermo-kinetic characterization of the as-prepared amorphous IMC. Note that the main challenge to overcome in the case of IMC is its high tendency towards thermal degradation, even during short-term storage [14]. A detailed description of the structural relaxation, macro- and microscopic crystal growth (and, as a consequence, also melting) and viscous flow processes of IMC is presented within the framework of the current research. In addition, mutual relationships between these phenomena are identified and discussed. The research was conducted with respect to the influence of the particle size of the powdered IMC to identify the ideal technological conditions for the processing and storage of IMC in the amorphous form.

## 2. Experimental Procedure

The melt-quench technique was used to prepare the bulk amorphous IMC from its pure γ-IMC polymorphic crystalline form (Sigma-Aldrich, Prague, Czech Republic). The applied procedure was as follows: (1) the heating of crystalline indomethacin in a glass vial (immersed in an oil bath) at a few degrees above the IMC melting point T_m_ ≈ 160 °C (note that the temperature of the oil bath was slowly increased until the first IMC crystals started to melt at the vial sides, and then the temperature was kept as constant until all the IMC melted); and (2) the quenching of the glass vial with the IMC melt in cold water (≈10 °C). The resulting product, the amorphous IMC, was pale yellow; this change in color is probably caused by the conjugation of the indole ring and the newly formed resonance structures in the amorphous form (i.e., the breach of the rigid interaction between the indole and benzene rings, which occurs in the crystalline form) [15]. Note that after the crystallization of the amorphous IMC (as described below), the crystalline form was again white, which indicates the reversible character of the indole/benzene electron interaction. The vitrified bulk IMC was powdered using an agate mortar and pestle and sieves (Retsch) with the following mesh sizes: 50, 125, 180, 250, 300 and 500 µm. A standard sieving procedure was implemented, with no additional pressure applied. Pieces of the amorphous IMC with a size of ≈1000 µm were denoted as the “bulk” samples. These samples were practically always measured in the form of a single piece of amorphous IMC matter, initially broken off from the main IMC ingot (in order to introduce the minimum amount of mechanically induced defects). The storage of the samples was conducted in a cooled (10 °C), dark desiccator. Since the IMC powders were found to degrade (nucleate) extremely quickly, even under these conditions, several batches of amorphous IMC were prepared under identical conditions, so that each sample was measured within 12 h of its preparation (the melt-quench formation of the bulk IMC ingot).

The manifestation of the macroscopic crystal growth was recorded calorimetrically, using a heat flow DSC instrument (Q2000, TA Instruments, New Castle, DE, USA) equipped with an autosampler, an RCS90 cooling accessory, and T-zero technology. The calibration of the DSC was realized using the In, Zn, and H_2_O standards. For the particle size (d_aver_) of each IMC powder, a set of DSC heating scans were performed in the 10–180 °C temperature range (with a defined particle size, as separated by the sieves). The following heating rates q^+^ were applied to each set: 0.5, 1, 2, 5, 10 and 20 °C·min^−1^. The fresh (as-prepared) sample was used for each heating scan, and the sample masses were approx. 2 mg (accurately weighted to 0.01 mg). The IMC samples were measured in hermetically sealed low-mass DSC pans. A similar experimental setup was also used for the structural relaxation measurements, where two types of temperature cyclic experiments were performed: the CR (constant ratio) cycles [16], with heating rates q^+^ being similar in the absolute magnitude to the preceding cooling rate q^−^, and CHR (constant heating rate) cycles [17], all at q^+^ = 10 °C·min^−1^. The cooling rates applied in the CR and CHR cyclic experiments were q^−^ = 0.5, 1, 2, 3, 5, 7, 10, 15, 20 and 30 °C·min^−1^. The reproducibility of both the crystallization and relaxation experiments (tested for several randomly selected measurements) was found to be very good if performed within a few hours of each other; however, longer delays resulted in slight temperature shifts and/or minor distortions of the corresponding kinetics.

The thermogravimetric characterization of the IMC decomposition was performed using the STA (TGA) 449 F5 Jupiter instrument (Netzsch, Selb, Germany) equipped with a DSC/TG holder. N_2_ was used as a purge gas at 50 mL·min^−1^. The TGA measurements were performed on approx. 2 mg samples (accurately weighted to 0.01 mg), placed in low-mass Al_2_O_3_ pans. The measurements were conducted as single heating scans of the amorphous samples in the 25–450 °C temperature range, and the applied heating rates were q^+^ = 0.5, 1, 2, 3, 5, 7, 10, 15, 20 and 30 °C·min^−1^.

An X-ray diffraction instrument (Empyrean Malvern Panalytical, Malvern, United Kingdom) was used to obtain the diffraction pattern of the DSC-crystallized IMC in the 5–50° range, and the XRD record was used to identify the main IMC polymorph formed during the DSC measurements. Similarly, the DXR2 Raman microscope (Nicolet, Thermo Fisher Scientific, Prague, Czech Republic), equipped with a 785 nm excitation diode laser (30 mW, laser spot size of 1.6 μm) and CCD detector, was used to collect the Raman spectra of the amorphous and DSC-crystallized samples. The experimental setup for the Raman measurements was a 25 mW laser power on the sample, 3s duration of a single scan, and 100 scans summed in one spectrum. The optical microscope iScope PLMi (Euromex, Arnhem, Netherlands), equipped with 40× and 80× high-quality objectives and a Moticam visual camera, was used in the transmission mode to photograph the typically formed crystallites.

## 3. Results

**Figure 1:**
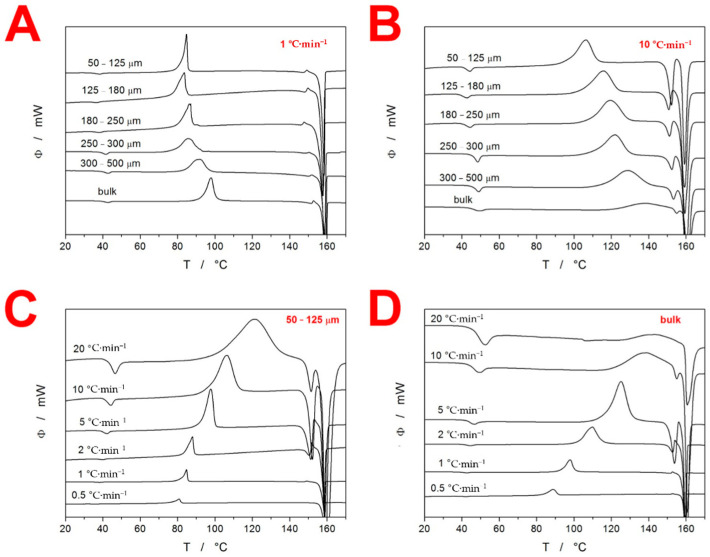
Example DSC curves obtained for the various fractions of IMC powders measured at selected heating rates q^+^. Exothermic effects evolve in the upwards direction: (**A**) all particle size fractions measured at 1 °C·min^−1^; (**B**) all particle size fractions measured at 10 °C·min^−1^; (**C**) 50–125 µm-particle size fraction measured at all q^+^; (**D**) bulk µm-particle size fraction measured at all q^+^.

The majority of the experimental data were obtained by means of differential scanning calorimetry. In Figure 1, several examples of the raw DSC curves (temperature dependence of the heat flow Φ) are shown, obtained for the powdered and bulk (single piece with a size of ≈1000 µm) IMC during the single heating scans under various conditions. The DSC curves show signals corresponding to three distinct physical transformations. At ≈40 °C, the endothermic step-like change of the heat capacity, interlinked with the structural relaxation peak, occurred. The relaxation peak is relatively prominent, clearly visible on the same scale as the dominant processes of crystallization and melting, which indicates that IMC is a relatively fragile material (as classified according to the Angell plot [18]). The second visible effect is the exothermic peak corresponding to the macroscopic manifestation of the crystal growth. Depending on the conditions (the average particle size d_aver_ and heating rate q^+^), the onset of the macroscopically observed crystallization occurred between 70–125 °C; this is a typical behavior of amorphous IMC prepared by the melt-quench technique. By comparison, the crystallization of amorphous IMC produced by the milling of the crystalline phase (not studied in the present paper) starts at ≈45–60 °C [19]. Apart from the preparation/synthesis route, the crystallization process is clearly largely dependent on the IMC form (powder particle size) and measurement conditions (applied q^+^). As is apparent from Figure 1A,B, the change in the powder size from 50–125 µm to approx. 1000 µm (assigned to the bulk pieces) caused a significant shift in the macroscopic kinetics: at low q^+^, the kinetics changed from the zero-order asymmetry [20] (≈ linearly ascending onset peak side and sharply descending end-set peak side) to the more or less symmetric peak characteristic of the nucleation-growth [21,22,23] or autocatalyzed n-th-order [24,25] kinetics; whereas at high q^+^, the nuances between the peak shapes were subtle, featuring slightly more positive asymmetries at larger particle sizes. This is, however, partially caused by the merging of the crystallization and melting effects. In addition to the changes in the peak shape, the increase in the powder size also slightly increased the temperature of the crystallization. This is especially important in the case of the coarsest powder and bulk IMC, where such an increase leads to the overlap of the crystallization and melting peaks.

The increasing particle size d_aver_ also indirectly, through the change in the crystal growth processes, influenced the melting peak of IMC. The melting of the DSC-crystallized IMC manifested as a double-peak at ≈149 and 157 °C, temperatures corresponding to the melting of the α-IMC and γ-IMC polymorphic crystalline forms, respectively. [26,27] In order to better visualize the melting peak onsets, the zoomed-in relevant temperature region is shown in Figure S1. With an increased particle size, the melting pre-peak (corresponding to the α-IMC polymorph) decreased in magnitude, which indicates lower amounts of the α-IMC phase being formed during the crystallization.

In Figure 1C,D, the influence of the heating rate q^+^ on the course of the DSC curves is depicted. The q^+^-based trends are qualitatively similar but significantly stronger compared to those caused by changes in particle size. The increase of q^+^ from 0.5 to 20 led to the shift of the increase in the glass transition temperature T_g_ by ≈ 10 °C, whereas the onset of the crystallization peak increased by ≈ 25–40 °C (depending on d_aver_). This indicates that the apparent activation energy was much higher for the glass transition phenomenon. The increase in q^+^ also drastically changed the peak asymmetries from highly negative ones to the almost symmetric peaks (this effect was more prominent for the low d_aver_). The most important feature associated with increasing q^+^ was the slowing down of the macroscopic crystallization, which, for the coarse powders and bulk particles (single pieces with a size of ≈1000 µm), caused the incomplete amorphous-to-crystalline transformation that was disrupted by the melting process-this was further reflected in the corresponding kinetic calculations. Further alterations to the crystallization process are reflected in the melting peak. The increasing q^+^ results in the rising portion of the crystalline phase being formed as α polymorph, which is dominant at a higher T [26,27]. This observation is valid up to the point where the crystallization starts to interfere with the melting, when the amount of the α polymorph in the overall crystalline phase decreases again.

**Figure 2:**
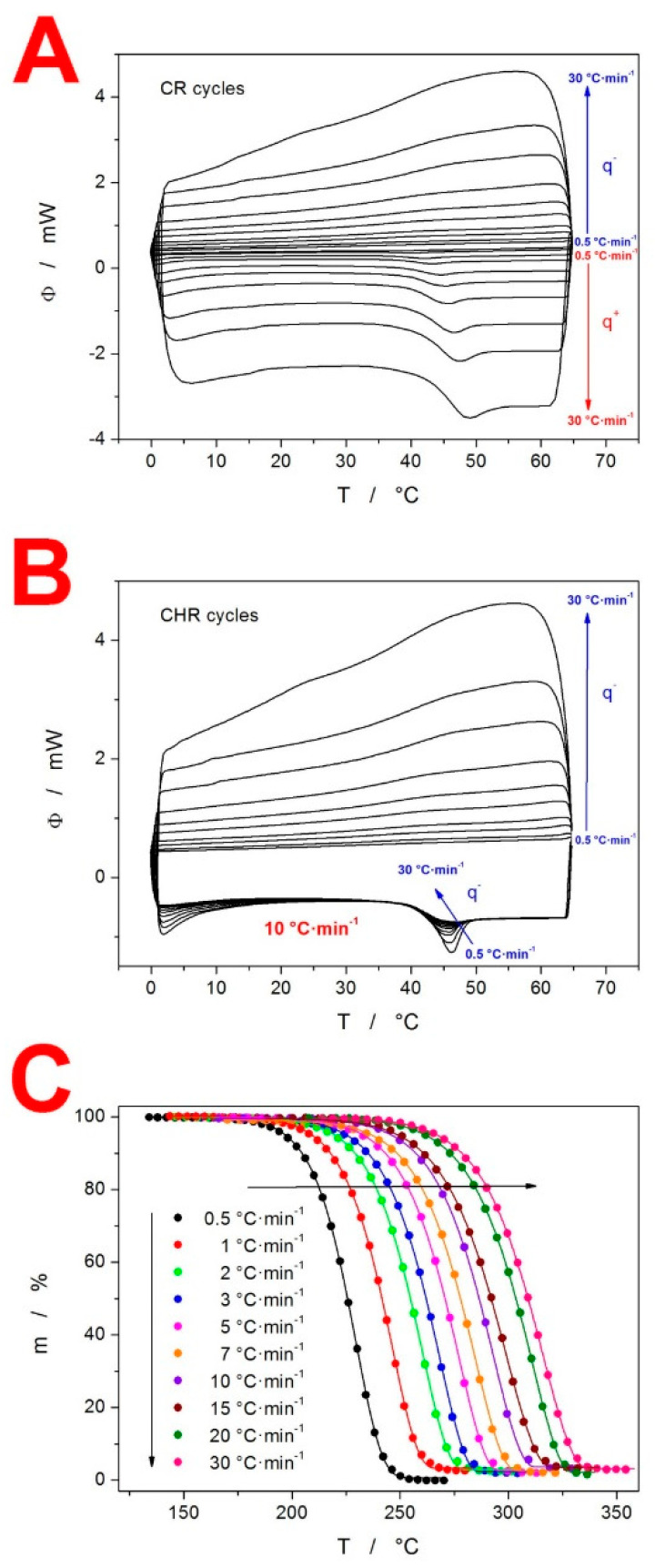
(**A**) Full DSC record of the set of CR cyclic relaxation measurements obtained for the amorphous IMC. The arrows and symbols q^−^ and q^+^ denote the parts of the DSC data in which the cooling and heating steps (respectively) of the CR cycles are shown. Absolute magnitudes of q^−^ and q^+^ applied in the corresponding steps of the cyclic program increase in the directions of the given arrows. (**B**) Full DSC record of the set of CHR cyclic relaxation measurements obtained for the amorphous IMC. In the CHR measurements, q^−^ varied, and q^+^ was always 10 °C·min^−1^. The arrow and symbol q^−^ denote the parts of the DSC data which differ in accordance with applied q^−^. In the upper part of the graph (where the cooling steps are depicted), the absolute magnitude of q^−^ increases with the arrow. In the part where the heating steps are shown, the arrow denotes the increase in |q^−^| in the preceding cooling step. (**C**) Full set of TGA curves obtained for the bulk IMC.

As stated in the introduction, the intended thermo-kinetic characterization of IMC includes (in addition to the crystallization process) a detailed investigation of the two other important kinetic phenomena: the structural relaxation and thermal decomposition. With regard to the former, in addition to the single heating scans, the DSC was also used to perform the CR and CHR cyclic relaxation experiments, as depicted in Figure 2A,B, respectively. In all cases, the relaxation peaks were well pronounced, with clearly identifiable peak maxima, as well as temperature dependences of the heat capacities (c_p_-T), in the glassy and undercooled liquid regions. Note that c_p_ is obtained by the normalization of Φ, and thus the temperature dependence of both quantities is technically similar (apart from the different units and scaling on the Y axis). Moreover, the very good reproducibility of the two c_p_-T dependences is demonstrated by the heating scans of the CHR cycles (see Figure 2B). In addition to the DSC, a second thermo-analytic technique, thermogravimetry (TGA), was utilized to characterize the prepared amorphous IMC and study its thermal decomposition. From a thermogravimetric point of view, IMC is stable up to ≈170 °C, where the decomposition begins in a single step at the slowest applied q^+^ = 0.5 °C·min^−1^. In Figure 2C, the complete set of TGA curves are displayed, showing the temperature shift at approx. 80 °C caused by the increase in q^+^ from 0.5 to 30 °C·min^−1^. Our TGA data are partially consistent with the results of Rusu et al. [28], who reported a similar temperature range for the decomposition process: 190–380 °C at q^+^ = 20 °C·min^−1^ (note that the slightly higher onset and end-set temperatures may be attributed to the high sample mass of ≈100 mg). However, whereas our TGA data show the practically complete decomposition and burnout/release of the decomposition products (with the residual masses ≤3%), Rusu et al. reported only a ≈ 65% mass loss in the first decomposition step, followed by the remaining mass being burnt/decomposed in a second separate step in the 420–720 °C range. This discrepancy may possibly be attributed to the fact that IMC was self-synthesized via a new method in [28], which might have resulted in some byproducts being present in the IMC sample subjected to the TGA measurements. A much stronger correlation was found between the present TGA data and the results of Tita at al. [29,30], who reported a single step decomposition in the 215–330 °C range (for q^+^ = 10 °C·min^−1^).

**Figure 3:**
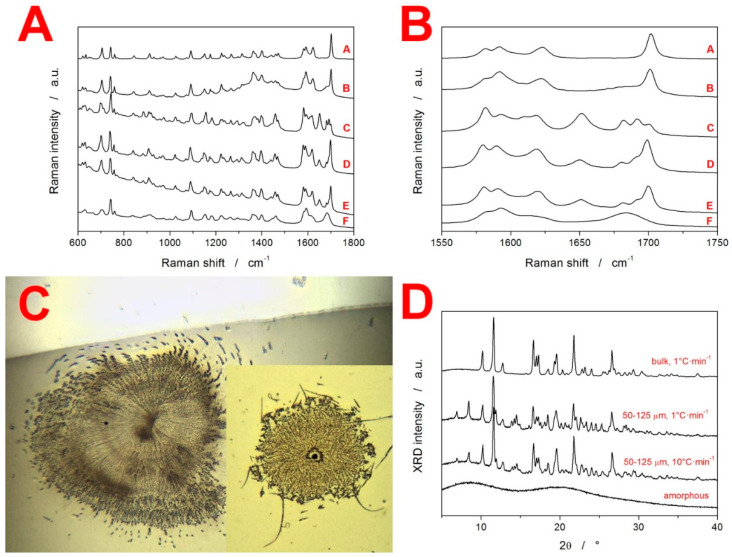
(**A**) Raman spectra for different types of IMC samples: A = original crystalline γ-IMC used to prepare the amorphous IMC, B = bulk IMC aged at room temperature for 48 h and DSC-crystallized at 10 °C·min^−1^, C = 50–125 µm IMC powder aged at room temperature for 48 h and DSC-crystallized at 10 °C·min^−1^, D = fresh 50–125 µm IMC powder DSC-crystallized at 1 °C·min^−1^, E = fresh 50–125 µm IMC powder DSC-crystallized at 10 °C·min^−1^, and F = amorphous IMC. (**B**) Raman spectra from graph A, zoomed-in on the 1550–1750 cm^−1^ range of the Raman shift. (**C**) Optical micrographs of the γ-IMC and possible τ-IMC (inset) crystals grown on the free bulk IMC surface. (**D**) XRD patterns obtained for the amorphous IMC, and IMC samples crystallized under various conditions.

In addition to the thermo-analytic measurements, several supplemental techniques were employed to characterize the as-prepared (amorphous) and DSC-crystallized IMC. The Raman spectra of the various IMC sample forms are displayed in Figure 3A, while the crucial range of the Raman shifts (based on the different IMC polymorphs that can be distinguished) is depicted zoomed-in in Figure 3B. Whereas the amorphous IMC is characterized by the broad Raman band at 1685 cm^−1^, γ-IMC is characterized by the 1700 cm^−1^ band (benzoyl C=O stretching), and α-IMC is characterized by bands at 1650 (benzoyl C=O stretching), 1680 (benzoyl C=O stretching) and 1692 cm^−1^ (acid O-C=O stretching). [27,31] The Raman spectra in Figure 3B indicate that the actual influence of q^+^ on the formation of the different polymorphs is rather minor, and we observed a similar representation of the α-IMC and γ-IMC in samples D and E (50–125 µm, q^+^ = 1 and 10 °C·min^−1^, respectively). The polymorphic preference is mainly determined by the long-term nucleation conditions: sample B (bulk-aged at room temperature for 48 h, then crystallized at 10 °C·min^−1^) crystallized into practically pure γ-IMC, whereas sample C (50–125 µm powder aged at room temperature for 48 h, then crystallized at 10 °C·min^−1^) exhibited the dominant representation of the α-IMC. The micrographs of the two types of crystallites occurring in the bulk samples are shown in Figure 3C. The main photograph shows the γ-IMC crystal (confirmed by Raman microscopy), and the inset depicts the possible τ-IMC crystal (Raman microscopy revealed a band at 1690 cm^−1^ but no band at 1650 cm^−1^, which is consistent with the τ polymorph [26]). Note, however, that these micrographs were obtained for the long-term low-T crystallization of the bulk sample. Distinguishing individual crystallites in the DSC-crystallized powders is not realistically achievable due to their high nucleation density and correspondingly large number of small irregular overlapping crystallites.

In Figure 3D, the XRD patterns are shown for the as-prepared amorphous IMC, 50–125 µm powders crystallized at different rates, and bulk sample aged at room temperature for 48 h and then crystallized at 10 °C·min^−1^ (note that this was the only way of producing pure γ-IMC due to the specific sub-T_g_ nucleation conditions and surface mobility, which were hindered by the presence of the mechanical defects, as explained in detail in Section 4.2). The characteristic diffraction lines for the γ-IMC are at 10.2, 11.7, 16.7, 19.6, 20.5 and 21.9°, while α-IMC has characteristic lines at 6.9, 8.5, 11.5, 11.9, 13.9, 14.2, 17.6 and 18.0 [26,27,32]. No polymorphs other than α-IMC and γ-IMC were obtained in the present study.

## 4. Discussion

In Section 3, the base characterization of the amorphous IMC was presented, together with the thermo-analytic curves reflecting the main kinetic processes occurring during heating of the glassy matrix. In the present section, the kinetics of these processes are described in terms of the state-of-art kinetic models (Section 4.1). Moreover, the mutual relationships between these processes are discussed in the case of IMC (Section 4.2).

### 4.1. Kinetics of the Thermo-Analytically Observed Phenomena

#### 4.1.1. Glass Transition Kinetics

**Figure 4:**
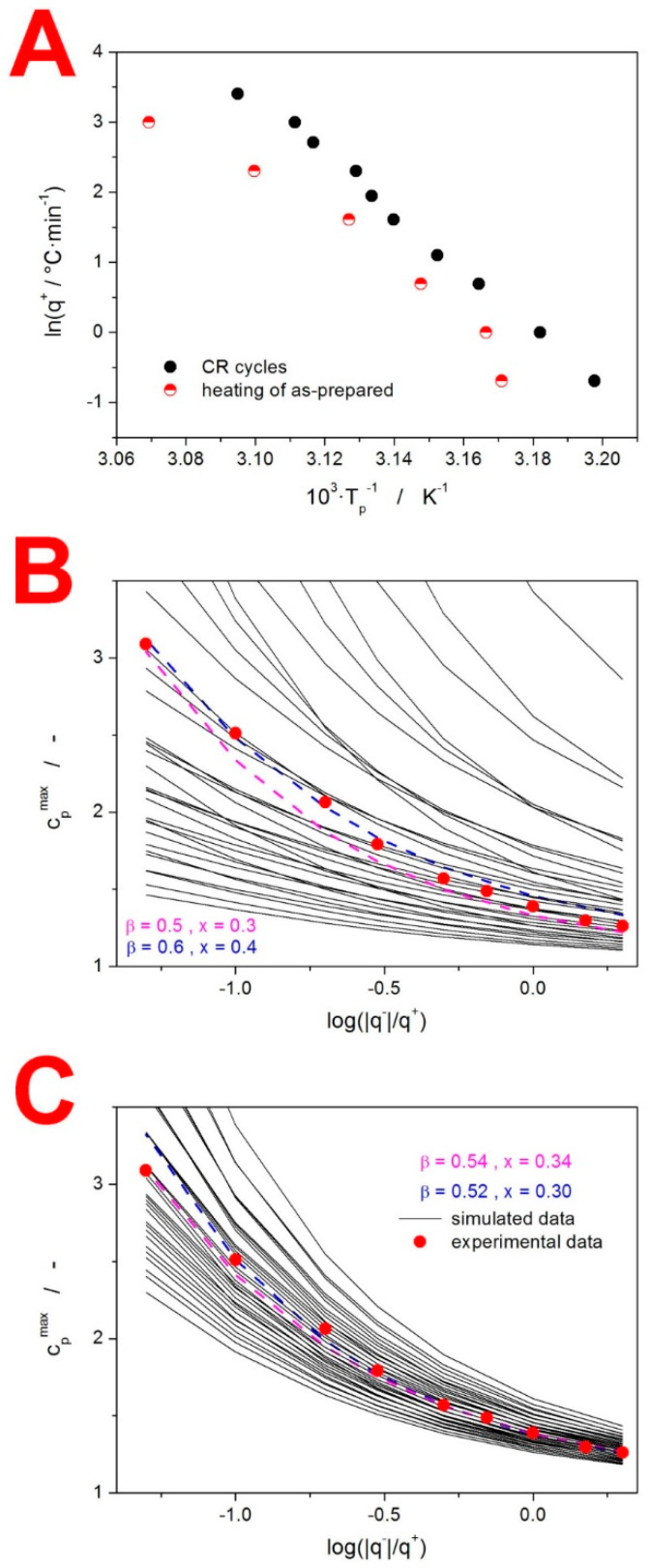
(**A**) Evaluation of ∆h* by applying Equation (3) to the CR cycles and to the single heating scans of the as-prepared IMC material (examples of these scans are shown in Figure 1). (**B**) Application of the simulation-comparative method to the CHR relaxation measurements of the amorphous IMC: 1st round. Experimental data points. Black solid lines refer to simulated data for the various combinations of the TNM parameters β and x (both parameters changing with the coarse step). Colored dashed lines refer to the two simulated β + x combinations best fitting the experimental data. (**C**) Application of the simulation-comparative method to the CHR relaxation measurements of the amorphous IMC: 2nd round. Experimental data points. Black solid lines refer to simulated data for the various combinations of the TNM parameters β and x (both parameters changing with the fine step). Colored dashed lines refer to the two simulated β + x combinations best fitting the experimental data.

Structural relaxation is the key phenomenon involved glass transition kinetics. Today, the structural relaxation process is usually described in terms of the phenomenological Tool-Narayanaswamy-Moynihan (TNM) model [33,34,35]:

(1) $$\Phi (t)=\exp\left[-{\left(\int_{0}^{t}\frac{\mathrm{dt}}{\tau \left({T,T}_{f}\right)}\right)}^{\beta }\right]$$

(2) $${\tau (T,T}_{f})=A_{\mathrm{TNM}}\cdot \exp\left[x\frac{{\Delta h}^{*}}{\mathrm{RT}}+(1-x)\frac{{\Delta h}^{*}}{{\mathrm{RT}}_{f}}\right]$$

where F_R_(t) is the relaxation function, defined as (c_p_ − c_pg_)/(c_pl_ − c_pg_), and c_pg_ and c_pl_ are the extrapolated heat capacities of the glass and undercooled liquid regions, respectively. Furthermore, t is time, τ is the relaxation time, β is the non-exponentiality parameter (0 < β ≤ 1), A is the pre-exponential factor, x is the non-linearity parameter (0 < x ≤ 1), ∆h* is the apparent activation energy of the structural relaxation, R is the universal gas constant, T is temperature and T_f_ is the fictive temperature. For the enthalpic manifestation of the structural relaxation recorded via calorimetric instrumental techniques, a procedural guide to the proper determination of the TNM parameters (∆h*, x, A, β) was published in [36]. In accordance with this practice, ∆h* should be determined from the CR cycles (see Figure 2A), according to [37]:

(3) $$-\frac{{\Delta h}^{*}}{R}={\left[\frac{\mathrm{dln}\left|q^{-}\right|}{d\left(1/T_{P}\right)}\right]}_{q^{-}/q^{+}=\mathrm{const}}$$

where T_p_ is the temperature corresponding to the maximum of the relaxation peak. This evaluation is presented in Figure 4A. Note that the strong linearity of this dependence confirms the absence of any significant (distorting) thermal gradients in the IMC samples for q^+^ up to 30 °C·min^−1^. The apparent activation energy determined from the CR cycle data, according to Equation (3), is ∆h* = 342 ± 7 kJ·mol^−1^.

Despite the ease, accuracy and robustness of this method, the current literature still often features the incorrect [38,39] evaluation of ∆h* using the heating of the as-prepared glassy samples with an undefined prior thermal history (measurements akin to those depicted in Figure 1). To demonstrate the fallacy of such an approach, the present data shown in Figure 1 were evaluated in this way, providing the curved dependences shown in Figure 4A. In addition to the ambiguous evaluation of ∆h* from this data (the apparent activation energy can vary between 190 and 1275 kJ·mol^−1^ depending on the evaluated q^+^ range), the ∆h* value determined for materials with an undefined thermal history does not behave as a material constant and depends on the prior thermal treatment [38,39].

With the knowledge of the ∆h* value, the curve-fitting approach [40] based on Equations (1) and (2) can be used to determine the pre-exponential factor: ln(A/s) = −127.35. In theory, a similar procedure can also provide the values of β and x. However, due to the instrumental artifacts and/or slight data distortions arising from the non-constant c_pg_-T and c_pl_-T dependences, the non-linear optimization method can only provide results that are not physically meaningful [36] (the curve-fitting of the present CHR cycle following cooling at 0.5 °C·min^−1^ resulted in x = 0.72 and β = 1). For this reason, a significantly more robust simulation-comparative method [41] is recommended. This method is based on the comparison of the experimental and theoretically simulated dependences of the height of the normalized relaxation peak c_p_^max^ during the CHR cyclic experiments (see Figure S2 for the visual demonstration of the c_p_^max^ determination). This evaluation of the present CHR data (see Figure 2B) is demonstrated in Figure 4B, where points correspond to the experimental c_p_^max^ values of the individual CHR cycles; black solid lines correspond to the theoretically simulated c_p_^max^-log(|q^−^|/q^+^) dependences for the combinations of β (ranging from 0.5–0.9 with the 0.1 step) and x (ranging from 0.3–0.9 with the 0.1 step); and color dashed lines correspond to the two best matches between the experimental and theoretically simulated data. Based on these results, a second round of simulations were performed for the combinations of β (ranging from 0.5–0.6 with the 0.02 step) and x (ranging from 0.3–0.4 with the 0.02 step) to improve the precision of their determination (see Figure 4C). This high-precision evaluation indicated that the TNM parameters for amorphous IMC should be β ≈ 0.53 and x ≈ 0.32. Note that the experimental data show a slightly non-monotonous trend in their dependence on log(|q^−^|/q^+^), hence the uncertainty of the evaluation.

#### 4.1.2. Crystallization Kinetics

The crystallization of the amorphous IMC was shown to exhibit significant dependences on both q^+^ and d_aver_ (see Figure 1). In order to quantify this behavior, the standard solid-state kinetic equation [42] was employed:

(4) $$\Phi =\Delta H\cdot A\cdot e^{-E/\mathrm{RT}}\cdot f\left(\alpha \right)$$

where Φ is the measured heat flow, ΔH is the crystallization enthalpy, A is the pre-exponential factor, E is the apparent activation energy of the macroscopic manifestation of the crystal growth, R is the universal gas constant, T is temperature and f(α) stands for an expression of a kinetic model, with α being the conversion. As the crystallization kinetics are significantly altered by the experimental conditions, a flexible autocatalytic Šesták–Berggren [24] (AC) kinetic function was chosen as the overarching model for the present study:

(5) $$f{\left(\alpha \right)}_{\mathrm{AC}}=\alpha^{M}{\left(1-\alpha \right)}^{N}$$

where M and N are the AC kinetic exponents of this empirical model. Furthermore, to demonstrate certain types of kinetic behavior, two additional model functions were used: the nucleation-growth Johnson–Mehl–Avrami [21,22,23] (JMA) model (Equation (6)) and zero-order [20] model (Equation (7)):

(6) $$f{\left(\alpha \right)}_{\mathrm{JMA}}=n\left(1-\alpha \right){\left[-\ln\left(1-\alpha \right)\right]}^{1-\left(1/m\right)}$$

(7) $$f{\left(\alpha \right)}_{F0}=1$$

where m is the JMA kinetic exponent. It is noteworthy that Equation (4) (in combination with any kinetic model, such as that introduced by Equations (5)–(7)) is a theoretical concept that describes only a single type of kinetic behavior. Any trends in the material form or experimental conditions (e.g., d_aver_ and q^+^) need to be quantified by introducing individual sets of parameters in Equation (4), corresponding to any specific combination of measurement conditions. In accordance with this concept, Equation (4) is enumerated for each d_aver_ (and technically also q^+^) separately, which allows one to identify the kinetic trends caused by both these experimental conditions. It is also important to mention that Equation (4) neglects the influence of pressure on the described kinetics, assumes a direct proportion between dα/dt and Φ, and anticipates the Arrhenian temperature dependence of dα/dt (which are all generally valid for the crystallization processes in the amorphous/glassy matrices).

**Figure 5:**
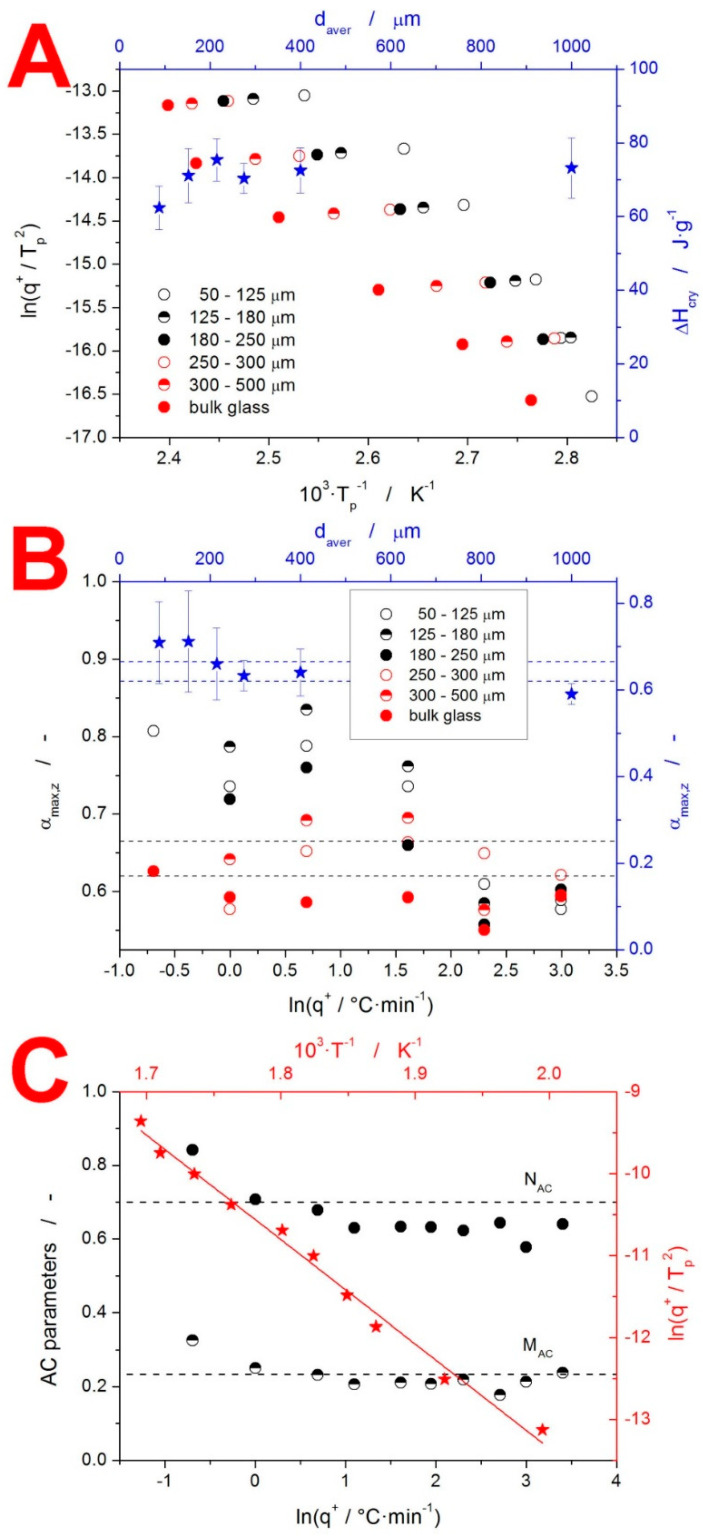
(**A**) Kissinger plot (constructed from the DSC crystallization measurements) for the different powder fractions of amorphous IMC. The right and top axes and star-shaped data points show the average crystallization enthalpy for each powder fraction (bulk samples were assigned as d_aver_ = 1000 µm). (**B**) Left and bottom axes show the q^+^ dependence of α_max,z_ for the crystallization measurements of the different IMC powder fractions. The right and top axes and star-shaped data points show the average α_max,z_ values for each powder fraction. The two pairs of horizontal dashed lines indicate the region of the JMA model applicability. (**C**) Left and bottom axes show the q^+^ dependence of the AC model parameters M and N, determined via sc-MKA for the decomposition of the IMC material measured by TGA. The dashed lines show the average M and N values obtained from the standard MKA. The right and top axes and star-shaped data points show the Kissinger plot constructed for the TGA decomposition data.

The enumeration of Equation (4) usually starts with the determination of the activation energy E. We recently showed that the original Kissinger equation [43] can be very beneficial (compared to, e.g., isoconversional methods [20]) in this regard. [44] The so-called Kissinger plot (the heating rate dependence of the temperature shift at the crystallization peak maximum T_p_; see Equation (8)) for the present IMC data is displayed in Figure 5A.

(8) $$\ln\left(\frac{q^{+}}{T_{p}^{2}}\right)=-\frac{E}{{\mathrm{RT}}_{p}}+\mathrm{const}$$

The linearity of the Kissinger dependence of the bulk IMC (single pieces with a size of ≈1000 µm) confirms that no distorting thermal gradients evolved within the sample or the DSC cell. Consequently, the curvatures observed for the IMC powders unambiguously indicate a change in the macroscopic crystal growth mechanism. Note that, in theory, such a change can be caused by a transformation of the native microscopic crystal growth mechanism [45]. However, as demonstrated below, in the case of IMC, the dominant influence on the macroscopic kinetics is most probably that of the nucleation and polymorphism. The linear fit of the dependences depicted in Figure 5A gives us the following apparent activation energies: 98 kJ·mol^−1^ (50–125 μm), 71 kJ·mol^−1^ (125–180 μm), 70.4 kJ·mol^−1^ (180–250 μm), 68 kJ·mol^−1^ (250–300 μm), 70.8 kJ·mol^−1^ (300–500 μm) and 72.5 kJ·mol^−1^ (bulk). It should, however, be noted that the only credible values are those of the two coarsest particle size fractions (300–500 μm powder and bulk), where the dependences are indeed linear. In the case of the finer powders, the rigorous procedure would be to determine the E-T dependence, as shown below. The second quantity to be enumerated in Equation (4) is the crystallization enthalpy ΔH. The particle size dependence of ΔH is shown in Figure 5A, averaging ≈72.5 J·g^−1^. The credibility of the ΔH data is supported by the simultaneous evaluation of the enthalpy of fusion ΔH_m_ = 105.2 J·g^−1^ (37.6 kJ·mol^−1^ at the molecular weight of IMC M_w_ = 357.8 g·mol^−1^), which is in perfect correspondence with the literature data [46]. With the knowledge that the IMC material subjected to the DSC crystallization measurements was fully amorphous, the discrepancy between ΔH and ΔH_m_ can be explained by Kirchhoff’s law (the temperature evolution of c_p_). Correspondingly, the lower ΔH in the case of the 50–125 μm powder fraction needs to be explained either by the different polymorph (the larger portion of α-IMC) or different crystallization mode (the dominant crystallization caused by mechanically induced defects), associated with a lower release of heat. Note that the simultaneous formation of the two polymorphic forms may be the main reason for the divergence of experimental data, even in the case of the isothermal measurements [47]. It is thus fully possible that this phenomenon is responsible for the changes in the crystallization kinetics in the case of the present non-isothermal measurements, where the impacts of the potential differences in the activation energy are augmented by the continuous increase in temperature.

As shown in Figure 1, the macroscopic crystallization kinetics showed great changes with both q^+^ and d_aver_. In such a case, the actual mathematically expressed physicochemical description of the kinetic data does not provide a transparent comparison of the kinetic behaviors. The suitable method of visualizing such data is the masterplot function z(α) [48], which for the non-isothermal data is defined as:

(9) $$z\left(\alpha \right)=f\left(\alpha \right)\cdot g\left(\alpha \right)\approx \Phi \cdot T^{2}$$

where g(α) is the integral form of the f(α) function (note that the right-hand side of Equation (9) is missing a negligible term that is omitted for practical purposes). The α value corresponding to the maximum of the z(α) function, α_max,z_, is the measure of the kinetic peak asymmetry characterizing the typical individual model behaviors. The α_max,z_ values for the present IMC crystallization data are shown in Figure 5B. The mean α_max,z_ values (averaged over the whole range of q^+^) show reasonable correspondence with the 0.632 fingerprint of the JMA model (the 0.999 correlation [49] with the JMA model is indicated by the dashed lines). This highlights, however, the danger of averaging data exhibiting significant trends. Therefore, the correct approach is to plot the individual α_max,z_ values as ln(q^+^) dependences (see Figure 5B). Based on this depiction, it is clear that only a small portion of the DSC curves correlate with the JMA model (as indicated by the dashed lines). For our IMC measurements, the α_max,z_ > 0.73 indicates the closing-in correspondence with the zero-order model, while the α_max,z_ < 0.60 indicates a higher-than-acceptable deviation from the JMA model, where the AC model should be used.

Therefore, the correct approach is to plot the individual α_max,z_ values as ln(q^+^) dependences (see Figure 5B). Based on this depiction, it is clear that only a small portion of the DSC curves correlate with the JMA model (as indicated by the dashed lines). For our IMC measurements, the α_max,z_ > 0.73 indicates the closing-in correspondence with the zero-order model, while the α_max,z_ < 0.60 indicates a higher-than-acceptable deviation from the JMA model, where the AC model should be used. Note that even the α_max,z_ range of 0.620–0.665 (for r^2^ = 0.999; or α_max,z_ = 0.585–0.705 for r^2^ = 0.995) does not guarantee the correspondence with the JMA model, and the direct curve-fitting should always be used to confirm the applicability of the JMA model. Considering the evolution of the α_max,z_ values in Figure 5B, the fine powders at low q^+^ exhibited a zero-order behavior (a slow growth from a large number of surface nuclei). With increasing d_aver_ and q^+^, the kinetic behavior changed through the nucleation-growth JMA-like kinetics to produce more symmetric peaks (describable only by the AC model). At the highest q^+^, all IMC powders crystallized with a more positive asymmetry than would correspond with the JMA model.

Since the present DSC data show a great variation in the peak asymmetries, the flexible AC model (capable of describing all the DSC kinetic curves) was used to provide the mathematical description of the thermo-analytic curves (utilizable for predictions of the macroscopic crystallization behavior). The single-curve multivariate kinetic analysis (sc-MKA) [50] was used for the curve-fitting of the crystallization data:

(10) $$\mathrm{RSS}=\sum_{j=1}^{n}\sum_{k={\mathrm{First}}_{j}i}^{{\mathrm{Last}}_{j}}w_{j,k}{\left({\mathrm{Yexp}}_{j,k}-{\mathrm{Ycal}}_{j,k}\right)}^{2}$$

(11) $$w_{j}=\frac{1}{{\left|{\left[d\alpha /\mathrm{dt}\right]}_{\max}\right|}_{j}+{\left|{\left[d\alpha /\mathrm{dt}\right]}_{\min}\right|}_{j}}$$

where RSS is the sum of squared residue, n is the number of measurements, j is the index of the given measurement, First_j_ is the index of the first point of the given curve, Last_j_ is the index of the last point of the given curve, Yexp_j,k_ is the experimental value of the point k of curve j, Ycal_j,k_ is the calculated value of the point k of curve j and w_j_ is the weighting factor for curve j. Note that Y_cal_ was modelled using Equations (4) and (5), while the standard MKA was used to determine E, which was then used as the fixed value for sc-MKA. [50] The kinetic parameters obtained by this procedure are listed in Table S1. The magnitude of the correlation coefficients for these descriptions indicates their practical usability in kinetic predictions of the IMC thermal stability (assuming that an accurate E-T dependence is correctly implemented).

The above-described behavior and trends in the crystallization kinetics can be used to interpret the raw data in Figure 1. However, additional information about the microscopic nucleation and crystal growth rates of particular IMC polymorphs [51] must also be considered and factored into the explanation: (1) below 55 °C, the nucleation of the γ form is dominant, while above this temperature, the α form of the IMC nucleates preferentially; and (2) above ≈50 °C, the crystal growth rate of the α form exceeds that of the γ form, and the difference between the two rates gradually increases with rising T. Starting with the influence of q^+^, the increase in the onset (as well as maximum) crystallization temperatures is inherently associated with the kinetics (activation energy, in particular). As a consequence of the main portion of the crystallization process occurring at a higher temperature, the larger amounts of α IMC are formed (due to both the shorter time spent nucleating below 55 °C and the greater difference between the crystal growth rates, favoring the α form being reached at higher temperatures), as evidenced by the melting peak proportions. The change in the peak asymmetry (from the zero-order-like model to the nucleation-growth and/or symmetric kinetics) can then be associated with the difference between the slow crystal growth from a high number of nuclei (low q^+^ and low T) and the fast crystal growth from a lower number of nuclei (fast q^+^ and high T). Note that additional changes in the crystallization peak asymmetry can be also caused by the increasingly greater overlap between the simultaneous formations of the two IMC polymorphs. With regard to the influence of d_aver_, the main factor is the effect of the presence of the mechanically induced defects that act as additional nucleation/crystallization centers, causing the crystallization behavior to become more reminiscent of the zero-order growth kinetics (growth from a large number of surface-located nuclei). In the case of the combination of high q^+^ and large d_aver_, the overall crystallization rate is so slow that the amorphous IMC transforms only partially into the crystalline form, when it has already reached the melting temperature T_m_. This not only slightly distorts the peak asymmetry but (most importantly) decreases the apparent ΔH. It is also noteworthy that the peak asymmetry is dominantly driven by q^+^, as both the IMC polymorphs exhibited nucleation-dominant crystallization.

#### 4.1.3. Decomposition Kinetics

The last thermo-analytically studied phenomenon was the decomposition of IMC. This process was independent from d_aver_ because it occurred in a high-T molten state (>50 °C above T_m_), where the material formed continuous fluid, regardless of its original solid-state form. As is often the case for thermal decompositions, the process kinetics were uniform, with the activation energy being almost constant and the kinetic model being consistent in all the experimental conditions. This is indicated by Figure 5C, where the Kissinger plot and results of the sc-MKA method are displayed. The evaluated activation energy for the decomposition process was 106 ± 4 kJ·mol^−1^. This value is in strong correspondence with the results of Tita et al. [29,30], who reported E values in the 105–120 kJ·mol^−1^ range (depending on the experimental conditions and evaluation methodology). The q^+^ dependence of the AC kinetic exponents M and N determined by sc-MKA (the points in Figure 5C) was very close to the overall values (horizontal dashed lines in Figure 5C) determined by standard MKA (the simultaneous fit of all the TGA curves combined), which indeed indicates the remarkable uniformity of the decomposition kinetics. It is, however, noteworthy that the AC kinetic exponents showed an increase at lowest q^+^, which is the crucial range of the experimental conditions for potential kinetic predictions.

### 4.2. Mutual Relationships between the Structural Relaxation, Crystal Growth and Viscous Flow

**Figure 6:**
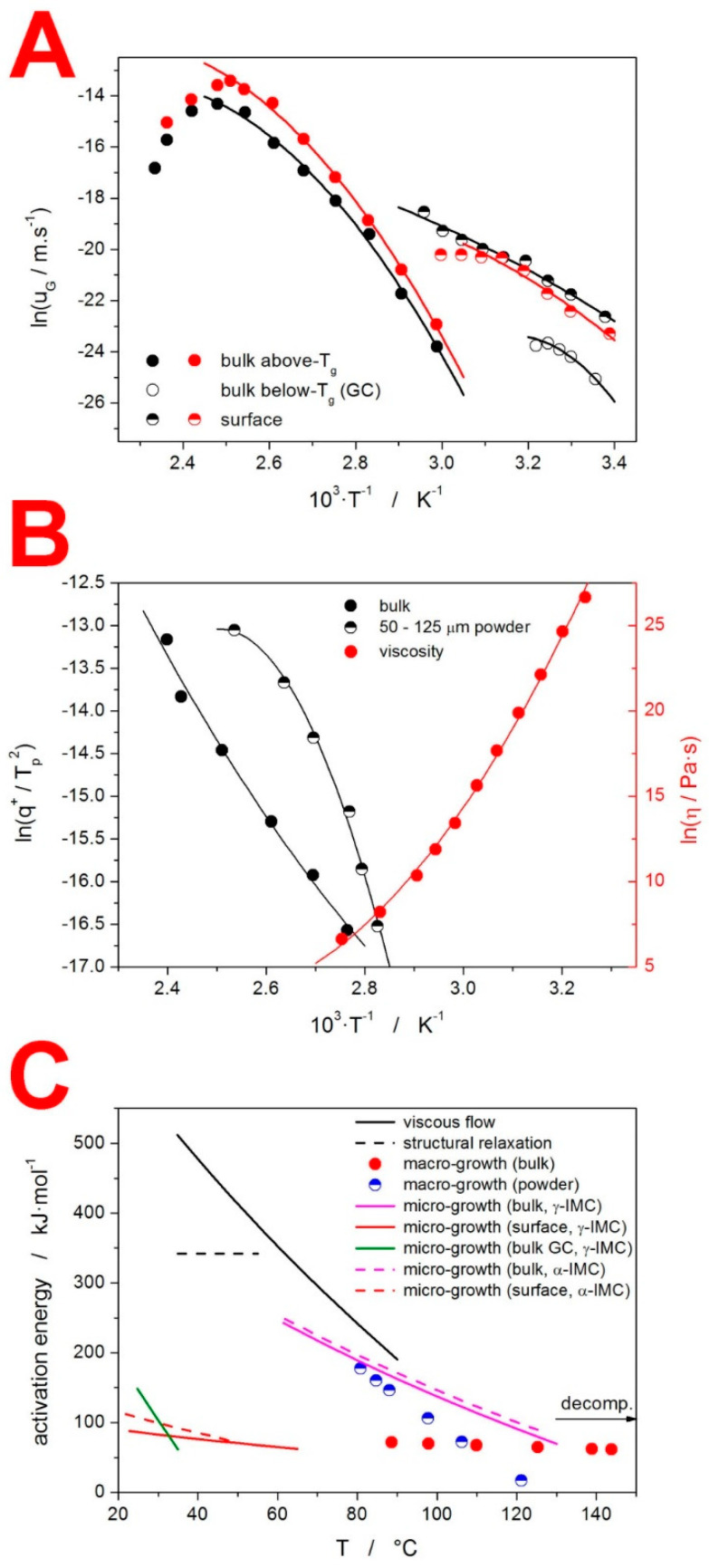
(**A**) Microscopic crystal growth rate data [13,54] fit by polynomial functions. Black-based points correspond to the γ form, and red-based points correspond to the α form. (**B**) Selected macroscopic crystallization data (in the form of the Kissinger plot, as depicted in Figure 5A, left axis) and viscosity data [55] (right axis) fit by polynomial functions. (**C**) Comparison of the temperature dependences of the activation energies in different processes occurring in the amorphous IMC. The magnitude of the decomposition activation energy is indicated by the horizontal arrow. The actual temperature range for this process is 170–330 °C.

As stated at the beginning of the present section, the mutual relationships between the key processes occurring in the amorphous IMC (structural relaxation, crystal growth and viscous flow) are discussed. The fundamental quantification of these relationships is based on the comparison of the activation energies in these processes. Since the absolute majority of these processes exhibit temperature dependent E values, we utilized the following dependences to determine the corresponding E-T data for the amorphous IMC: a Kissinger plot (Equation (8)) for the macroscopic crystal growth; the temperature dependence of the crystal growth rate u_G_ (Equation (12)) [52] for the microscopically observed crystal growth; and the temperature dependence of the viscosity η (Equation (13)) [53] for the activation energy of the viscous flow:

(12) $$\frac{\mathrm{dln}\left(u_{G}\right)}{d\left(1/T\right)}=\frac{E}{R}$$

(13) $$\frac{\mathrm{dln}\left(\eta \right)}{d\left(1/T\right)}=\frac{E_{\eta }}{R}$$

where the crystal growth rate u_G_ is defined as dr/dt (r standing for the crystal radius). The u_G_-T data were taken from [13,54], where optical microscopy was used to extensively study the crystal growth in γ-IMC and α-IMC, both above and below T_g_, in bulk and on the surface. The η-T data were taken from [55]. The above-mentioned temperature dependences are displayed in Figure 6A,B, together with their polynomial fits (used to interpolate the dependences and calculate the derivations according Equations (8), (12) and (13)). The corresponding E-T dependences are shown in Figure 6C, which also includes the apparent activation energy of the structural relaxation ∆h*, the temperature range of which corresponds to the span of the T_g_ values in Figure 2A. The value of the activation energy in the decomposition process is, in Figure 6C, indicated by the horizontal arrow. The actual temperature range used for this process should be 170–330 °C (see Figure 3C).

One of the aims of the present paper was to review the particular relationships between the three key processes that are interlinked in every glassy matrix: the viscous flow, structural relaxation and crystal growth. Starting with the connection between the viscosity and structural relaxation, this link has long been believed to be based on the similarity of the activation energies between the two processes [56,57,58]. This concept seems to be not valid in the case of IMC; compared to ∆h* ≈ 340 kJ·mol^−1^, the activation energy of the viscous flow E_η_ varied between 380 and 520 kJ·mol^−1^ in the corresponding temperature range. An obvious cause for such a discrepancy might be the microscopic crystal growth on the surface of the bulk samples (the viscosity measurements were performed by means of thermo-mechanical analysis), which would significantly increase the η values. However, the authors in [55] stated that, before and after the measurements, the samples were examined by polarized microscopy, and no traces of crystalline phase were found.

The further quantification of the relationship between the structural relaxation and viscosity can be understood in terms of the kinetic fragility concept [18], defined in Equations (14) and (15):

(14) $$m_{\mathrm{visc}.}={\frac{\mathrm{dlog}\eta }{{d(T}_{g}/T)}|}_{T=T_{g}}≅\frac{E_{\eta }/R}{T_{g}\ln(10)}$$

(15) $$m_{\mathrm{DSC}}={\frac{\mathrm{dlog}\tau }{{d(T}_{g}/T)}|}_{T=T_{g}}≅\frac{{\Delta h}^{*}/R}{T_{g}\ln(10)}$$

The present IMC data give m_visc._ = 89.6 and m_DSC_ = 56.7, again indicating a marked difference between the two processes. Note that the materials typically labelled as strong and fragile exhibit fragilities of 20 and 80, respectively (the theoretical limits for m are approx. 16–200) [18].

The second mutual relationship to discuss is that between the microscopic and macroscopic manifestations of the crystal growth in the amorphous IMC (with a particular focus on the thermodynamic/kinetic preferences of the individual polymorphs). Here, we argue, in general consensus with the recent literature, that the key process of the macroscopic manifestation of the crystal formation is actually the nucleation, and not the crystal growth itself. It is the nucleation conditions that largely determine the macroscopic outcome recorded by, e.g., calorimetric techniques. The activation energy barrier ΔG* for the nucleation process is (according to the classic nucleation theory [59]) determined as:

(16) $${\Delta G}^{*}=\frac{16\pi \sigma^{3}}{3\Delta G_{V}^{2}}$$

where σ is the interfacial energy (positive in the newly created/growing phase) and G_v_ is the difference between the Gibbs energies of the amorphous and crystalline phases (negative with the increasing volume of thermodynamically more stable phase). In general, the metastable polymorphs have lower σ (which is also the case for α-IMC, as compared to γ-IMC [60]), which can result in their kinetically preferential formation in cases of high molecular mobility. [26] Accordingly, a slow crystallization favors thermodynamically stable crystalline forms, whereas a rapid transformation will produce metastable polymorphs. [61] This is clearly the case of IMC, as evidenced in Figure 1, where, at a higher q^+^, the melting pre-peak of α-IMC is more prominent. Similar results, featuring a more positive asymmetry of the DSC crystallization peak and formation of the α-IMC after the rapid cooling of the melt (producing a loose glassy structure with internal voids), were reported in [14]. The above-discussed interplay between the interfacial energy and Gibbs energy as a function of molecular mobility has the characteristics of homogeneous nucleation. However, for amorphous materials, near T_g_, (and small-molecule organic glasses, in particular), the heterogeneous nucleation mechanism is dominant, which is clearly shown in Figure 1, where the presence of mechanically induced defects [62] favored the formation of the metastable α-IMC polymorph via the selective lowering of the crystallization activation energy barrier. Note that the crystal growth (micro- and macroscopic) in the amorphous IMC was initiated solely by either the surface or internal defects (micro-cracks, scratches, abrasions, etc.). This is clearly evidenced by numerous literature data, in studies where either the high stability of bulk IMC was reported [13,63,64], or the measurable crystal growth (bulk, surface and rapid GC growth) was reported for the conditions of crystal growth on the non-native (altered/damaged) surface or along the internal cracks [13,64,65,66,67,68]. This statement is also supported by our own tests, where we observed no crystal growth in the IMC droplet (the native surface formed during the free cooling of the melt) at 30 °C over 60 days, whereas similar droplets with even very slightly damaged surfaces (small scratches, equating to a light touch with a needle or gentle rub with cotton), under similar conditions, exhibited the rapid development (clearly detectable within 2 days) of crystallites in the damaged regions. This clearly underlines the importance of nucleation-focused studies (as opposed to the presently popular topic of microscopically studied crystal growth) and papers dealing with the effects of mechanical defects and stresses [69,70], internal stresses caused by tension (either positive or negative pressure formed due to the plasticization and formation of voids during crystal growth, or differences between thermal expansion coefficients) [71], and glass formation conditions.

The complexity of the macroscopic crystallization process in the case of low-molecular organic glasses was excellently defined in [72] as the interplay between four aspects: “(1) thermodynamics is the driver; (2) molecular mobility is the facilitator; (3) interface energy is the modulator; (4) heterogeneities and cracks are amplifiers.” Based on the dominant type of the crystallization center/nucleus, a number of crystallization profiles can develop in the macroscopic (e.g., calorimetry) record. Example types of these crystallization centers are homogeneous nucleation in the volume, heterogeneous nucleation in the volume, heterogeneous nucleation along the internal cracks, secondary volume nucleation on the interface between the crystalline and amorphous phases (including the external seeding), heterogeneous nucleation on the surface, heterogeneous nucleation due to mechanically induced defects and non-diffusional glass-crystal (GC) growth [59,62,66,68]. As is apparent, the majority of these nucleation mechanisms involve or require the presence of some defects in the amorphous matrix. The consequent crystal growth (growth of the initially formed nucleus) can then be either accelerated, hindered or not influenced by the presence of these defects. Whereas the above discussion downplayed the importance of the absolute values of the crystal growth in the macroscopically observed crystallization, there were still significant correlations between the activation energies of the two processes (as shown in Figure 6C). The activation energies of the microscopic crystal growth (evaluated from the data [13] in Figure 6A) were markedly similar for both IMC polymorphs, as were the absolute values of u_G_ [13]. At lower temperatures (≈80 °C), the E_G_ values for the bulk sample were close to the macroscopic E values of the powdered IMC. At higher temperatures (≈130 °C), the E_G_ values for the bulk sample approached the E values of the bulk IMC. To offer a hypothetical explanation for this behavior, we must take into account the sample preparation for the bulk microscopic measurements, as reported in [13]: the IMC was melted between two parallel glass coverslips, and after quenching the system, the upper coverslip was carefully removed. Such a procedure inevitably creates surface corrugation, which can result in accelerated nucleation and/or hindered crystal growth (due to possible micro-cracks and other obstacles positioned along the growth path). At low temperatures, the surface remains as formed during the coverslip removal, and the higher number of surface defects may lead to higher E_G_ (≈200 kJ·mol^−1^) as a result of the steeper u_G_-T dependence. This translates into a similar situation as that observed in the case of the powdered IMC. At higher temperatures, the lower η may enable a faster rearrangement and self-healing of the glass surface, and the E_G_ moved closer to the E of the calorimetrically studied bulk sample (≈65 kJ·mol^−1^), where a similar native surface was present. Another important correlation is that between the E_G_ obtained from the free surface and the calorimetric E obtained from the bulk sample. The similarity between the two activation energies suggests the continuity of the two processes between the microscale and the macroscale (again, since the surface growth was much quicker than it was in the bulk IMC, very similar types of nucleation and surface growth processes must have been present in the bulk glass subject to the calorimetric investigation).

The third mutual relationship to briefly discuss is that between the viscosity η and microscopically observed crystal growth rate u_G_, and the possible violation of the Stokes–Einstein law [10]. This correlation is widely discussed in the literature in terms of the Ediger’s decoupling parameter ξ [12]:

(17) $$\xi =\frac{d\left[\log\left(u_{r}\right)-f_{p}\right]}{\mathrm{dlog}\left(\eta \right)}=\frac{d\left[\log\left(u_{r}\right)-\log\left(1-\exp\left(-{\Delta G}_{\mathrm{lc}}/\mathrm{RT}\right)\right)\right]}{\mathrm{dlog}\left(\eta \right)}=\frac{\mathrm{dlog}\left(u_{\mathrm{kin}}\right)}{\mathrm{dlog}\left(\eta \right)}$$

where f_p_ is the probability of the structural entity (newly attached to the crystal growth interface) remaining in the crystalline state, and ΔG_lc_ is the difference between the Gibbs energies of the undercooled liquid and crystalline phases. The log(u_kin_)-log(η) dependence constructed using the literature data [54,55] is included in Figure S3, giving the data yield ξ = 0.683–0.695 (depending on the evaluation method, as discussed therein), which is consistent with the reports utilizing, e.g., dielectric measurements [66,73,74]. In his original derivation of the decoupling parameter ξ [12], Ediger demonstrated the correlation between ξ and the kinetic fragility m (ξ decreases with increasing m). These findings were revisited and refined by Nascimento et al. [75], who implemented additional crystal growth models (screw dislocation and 2-dimensional surface-nucleated models, as additions to the normal growth model used originally [12]) in the u_kin_ calculation. This correction restored the ability of the viscosity to describe the transport element of crystal growth rate in the η < 10^6^ Pa·s range. For strong glasses, no violation of the Stokes-Einstein law was observed up to η ≈ 10^12^ Pa·s. However, a certain u_kin_-η decoupling was still identified in the fragile glasses at high viscosities (low temperatures nearing T_g_). An alternative revision of the u_kin_-η decoupling was provided by Schmeltzer et al. [76], who introduced a decoupling temperature T_d_, below which the decoupling between the diffusion and viscosity occurs, with the decoupling exponent being temperature dependent (a qualitatively similar finding was recently obtained for amorphous selenium [77]). An interesting alternative to the above-mentioned standard models for crystal growth is the approach of Martin et al. [78], who introduced the “transition zone theory” based on the cooperating ensemble of structural entities driving the mechanistic formation of the crystalline phase.

The concept of cooperating regions (as introduced in [78] for the description of the crystal growth) can also qualitatively be applied to explain the fourth mutual relationship in the present discussion: that between the structural relaxation and crystal growth. Over the past years, such a correlation has been suggested based on the structural behavior related to the distribution of the relaxation times (expressed by β in the TNM model) within the concept of cooperatively rearranging regions [79]. Note that the determination of the distribution of the relaxation times is usually achieved by means of dielectric relaxation measurements in the T_x_–T_g_ temperature region (T_x_ standing for the crossover temperature, where the main α relaxation and the secondary Johari-Goldstein (JG) relaxations decouple [80]). In the case of IMC, the JG relaxations are believed to be the one of the primary mechanisms of the initiation of the nucleation and crystal growth. [65,72,81] Although the actual magnitude of this effect is still disputed, it has been shown [82] by the molecular dynamic simulations that the product D·τ_α_ (D being the self-diffusion coefficient, and τ_α_ being the structural relaxation time) drastically changes with even very slight changes in the distribution of the relaxation times (which are the main concern of this theory). These simulations have also unambiguously correlated the evolution of D·τ_α_ with the spatially heterogeneous dynamics [83,84]. Further models interlinking the structural relaxation kinetics and the surface mobility responsible for, e.g., the fast surface crystal growth of GC growth are listed in [67]. Whereas the concept of cooperating regions (as described by various structural relaxation models) is generally considered as the essential condition for at least certain crystal growth modes, no general consensus has been reached regarding the suitable quantification of this behavior. Here, we intend to propose two ideas based on the interpretation of the material’s tendency towards structural cooperativity between the below-T_g_ and above-T_g_ temperature regions. Firstly, the TNM parameter β, expressing the distribution of the relaxation times, does not necessarily need to be directly correlated with the structural cooperativity, as it only indicates the degree of qualitative variety in the structural domains. It is, in fact, the higher values of β (corresponding to the narrow distribution of τ_α_) that may indicate the higher cooperativity. In addition, the correlation between β and the effective cooperativity (with the greatest contribution to the initiation of the crystal growth) does not need to be monotonous. An extreme (maximum) form of this dependence might occur with reasonably high β values, where a rather narrow distribution of τ_α_ may correspond with the glassy state containing large cooperating domains surrounded by a higher energy microstructure. The cooperative characteristics of such a state could be associated with the size and packing of the domains, as well as with the fluidity and thickness of the separating high-energy layers. The second proposed idea is that the actual cooperativity of the glassy regions may also (if not better) be expressed by the TNM non-linearity term (1 − x), which defines the dependence of the structural relaxation motions on the actual material’s structure (in contrast to the dependence on T). The significant u_kin_-η decoupling identified in IMC may be a consequence of the relatively low value of x_TNM_ ≈ 0.32, which indicates the high cooperativity of the glass structural units and domains during the relaxation movements.

## 5. Conclusions

The thermo-kinetic behavior of amorphous IMC was studied by means of DSC, TGA, XRD and Raman microscopy, and the processes of structural relaxation, crystal growth and decomposition were investigated. The structural relaxation (kinetics of the glass transition phenomenon) was described in terms of the TNM model with the following parameters: ∆h* = 342 ± 7 kJ·mol^−1^, ln(A/s) = −127.35, x = 0.32 and β = 0.53. The importance of the defined glass thermal history for the evaluation of ∆h* was demonstrated. The activation energy of the structural relaxation was found to be significantly lower than the activation energy of the viscous flow in the corresponding temperature region.

The calorimetrically recorded macroscopic manifestation of the crystal growth exhibited dramatic changes in kinetics (as well as the polymorphic representation) in terms of q^+^, d_aver_ and aging (time of storage). Increasing q^+^ (and, to a smaller extent, increasing d_aver_) led to a change from zero-order kinetics (characterized by a large number of surface nuclei and continually inward-growing compact crystalline layer) to a more nucleation-growth-balanced symmetry with elements of autocatalytic behavior. Moreover, higher q^+^ resulted in a considerable amount of α-IMC being formed during crystallization, whereas at low q^+^, the γ polymorph was entirely dominant. The prolonged storage or processing of amorphous IMC (delays >24 h between the synthesis/quench and measurements) resulted in a marked acceleration of the DSC-measured crystallization. All these findings indicate the crucially prevailing influence of the nucleation on the overall crystallization process. The selective formation of the particular IMC polymorphs also confirms that slow crystallization favors thermodynamically stable crystalline forms, whereas a rapid transformation produces metastable polymorphs. A strong correlation was found between the microscopically determined activation energy of the surface crystal growth and the macroscopically obtained E pf the bulk samples (single pieces with a size of ≈1000 µm), with both energies showing a T-resolved concurring trend within the range of 60–90 kJ·mol^−1^ over ΔT ≈ 120 °C (indicating that mechanistically identical processes were involved).

Based on the literature data for the viscosity and microscopic crystal growth, a discussion of the evaluation of Ediger’s decoupling parameter ξ was provided in terms of the uncertainties associated with the extrapolations of the u_kin_-T and η-T dependences. Regarding the influence of the structural relaxation movements on the crystal growth process, the ability of the TNM relaxation model to express the degree of cooperativity in the glassy structure (that may be translated into the material’s behavior above T_g_) was discussed. In particular, the potential importance of the non-linearity parameter x (associated with the influence of the structural packing on the relaxation movements) was stressed.

## Data Availability

The original data are available on request from the corresponding author.

## Supplementary Materials

The following supporting information can be downloaded at: https://www.mdpi.com/article/10.3390/molecules27175668/s1, Figure S1: Zoomed-in melting region of the DSC curves; Figure S2: Schematic representation of the determination of the C_p_^max^ quantity; Figure S3: Evaluation of the decoupling parameter ξ; Table S1: Parameters of the standard kinetic equation.

## Sample Availability

Samples of the IMC compound are available from the authors on request.
