# Supplementary material for: Indomethacin: The Interplay between Structural Relaxation, Viscous Flow and Crystal Growth
Source: Molecules. 2022 Sep 2;27(17):5668. doi: 10.3390/molecules27175668 (PMC9458118; doi:10.3390/molecules27175668)
Supplement: Supplementary file 1 [file molecules-27-05668-s001.zip › molecules-1854788-supplementary.pdf]

Part 1

Zoomed-in temperature region of IMC melting originally displayed in Figs. 1A and 1B.

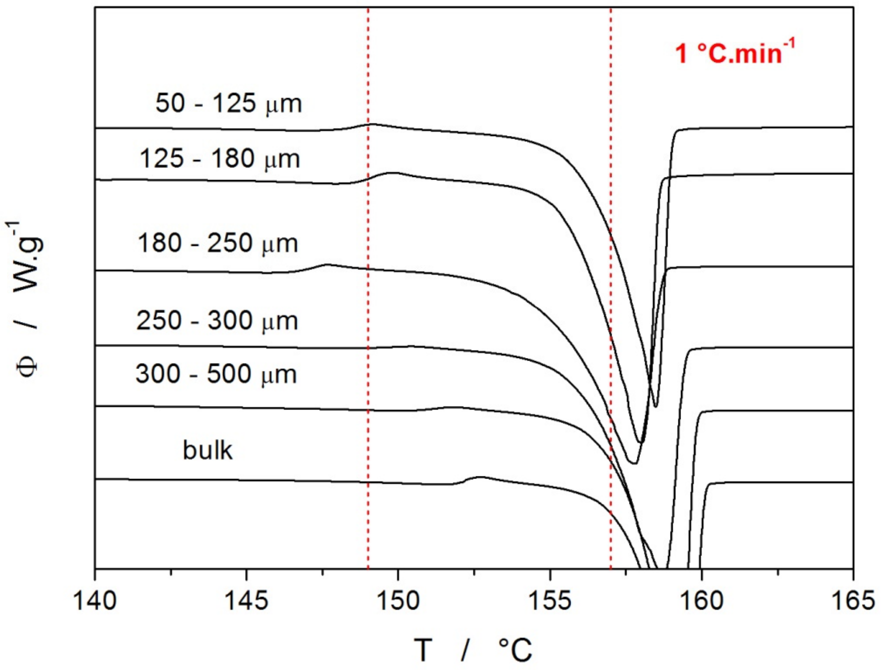

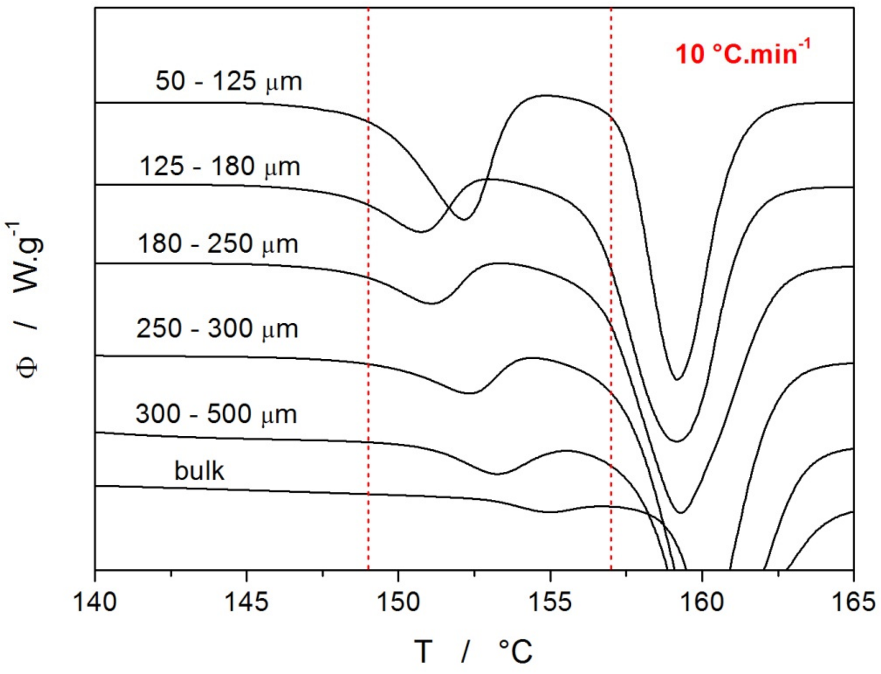

Figure S1: Zoomed-in melting region of the DSC curves.

Part 2

Schematic representation of the determination of the C_p^{max} quantity.

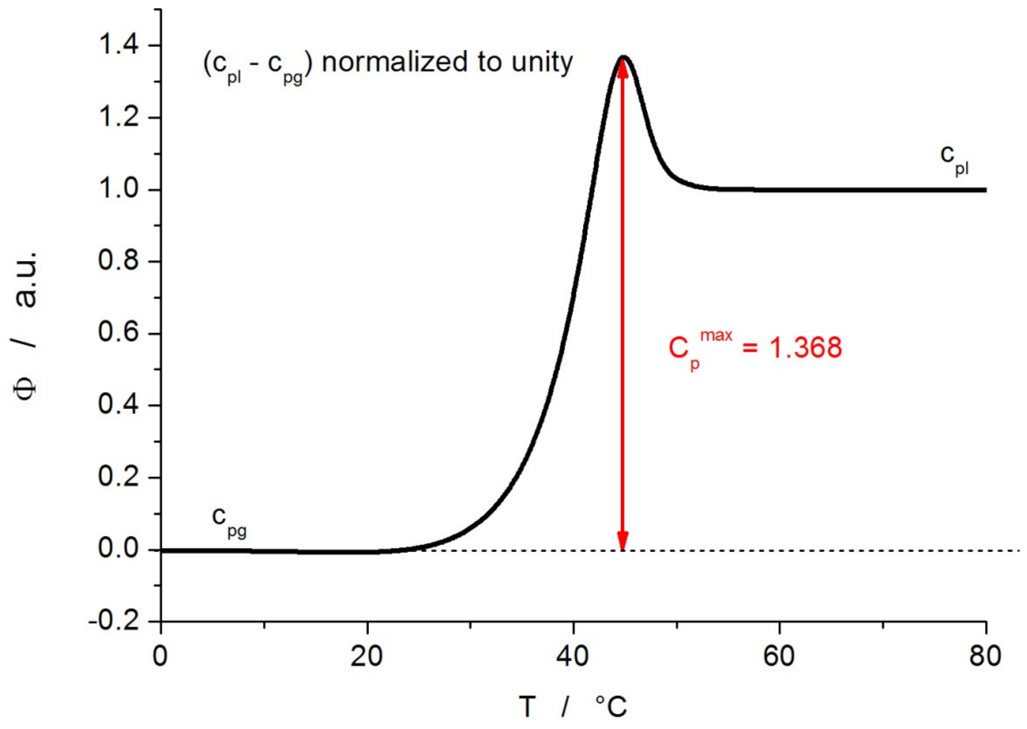

Figure S2: Schematic representation of the determination of the Cpmax quantity.

Part 3

Parameters of the standard kinetic equation (Eq. 4) coupled with the AC kinetic model (Eq. 5) enumerated by means of the sc-MKA method. Correlation coefficients for the individual fits are listed as well.

Table S1: Parameters of the standard kinetic equation.

	**50-125 µm**					
**q^+ / °C.min^{-1}**	0.5	1	2	5	10	20
log(A/s)	12.1551	12.48222	12.50363	12.44423	12.18019	11.66115
E / kJ.mol^{-1}	98	98	98	98	98	98
N	0.42086	0.41443	0.41891	0.55071	0.80383	0.99412
M	0.82346	0.91665	0.79913	0.81817	0.66534	0.42489
correl. coeff.	0.982656	0.980678	0.991774	0.997461	0.99838	0.99973
	**125-180 µm**					
q^+ / °C.min^{-1}	0.5	1	2	5	10	20
log(A/s)		8.36231	8.73434	8.5309	8.12952	7.91688
E / kJ.mol^{-1}		71	71	71	71	71
N		0.52278	0.39552	0.54293	0.76442	0.97683
M		0.8527	0.97482	0.89585	0.70439	0.59205
correl. coeff.		0.989558	0.976674	0.99396	0.999638	0.999565
	**180-250 µm**					
q^+ / °C.min^{-1}	0.5	1	2	5	10	20
log(A/s)		8.13746	8.52425	8.31951	7.94995	7.74163
E / kJ.mol^{-1}		70.4	70.4	70.4	70.4	70.4
N		0.46246	0.42447	0.70531	0.85503	0.93515
M		0.82928	0.96725	0.85936	0.69348	0.59279
correl. coeff.		0.986873	0.989898	0.996875	0.999698	0.997615
	**250-300 µm**					
q^+ / °C.min^{-1}	0.5	1	2	5	10	20
log(A/s)		7.67019	7.92241	7.81207	7.56151	7.38399
E / kJ.mol^{-1}		68	68	68	68	68
N		0.86792	0.61421	0.61919	0.76702	0.87189
M		0.7781	0.86982	0.79805	0.70793	0.55384
correl. coeff.		0.998365	0.997017	0.996695	0.99766	0.999619
	**300-500 µm**					
q^+ / °C.min^{-1}	0.5	1	2	5	10	20
log(A/s)		7.9285	8.12453	7.88471	7.70886	7.61467
E / kJ.mol^{-1}		70.8	70.8	70.8	70.8	70.8
N		0.85712	0.60012	0.63587	0.98552	0.93667
M		0.76479	0.85589	0.76074	0.66244	0.56131
correl. coeff.		0.999123	0.997581	0.996006	0.999686	0.996999
	**bulk**					
q^+ / °C.min^{-1}	0.5	1	2	5	10	20
log(A/s)	8.08075	8.24476	8.04987	7.95297	7.62333	7.8098
E / kJ.mol^{-1}	72.5	72.5	72.5	72.5	72.5	72.5
N	0.61426	0.78373	0.97222	0.82658	1.28275	0.8439
M	0.78548	0.89776	0.81464	0.80578	0.60055	0.52237
correl. coeff.	0.997558	0.993787	0.999013	0.995745	0.99688	0.994962

Part 4

Evaluation of the decoupling parameter ξ taken from the literature data [52, 53]. Considering the precision of the u_G and η fits depicted in Figs. 6A and 6B, we assumed that the extrapolation of the u_G-T dependence at lower temperatures was more accurate than the extrapolation of the η-T dependence at the higher temperatures. The correlation of the u/u_{kin} with η was thus constructed in this way.

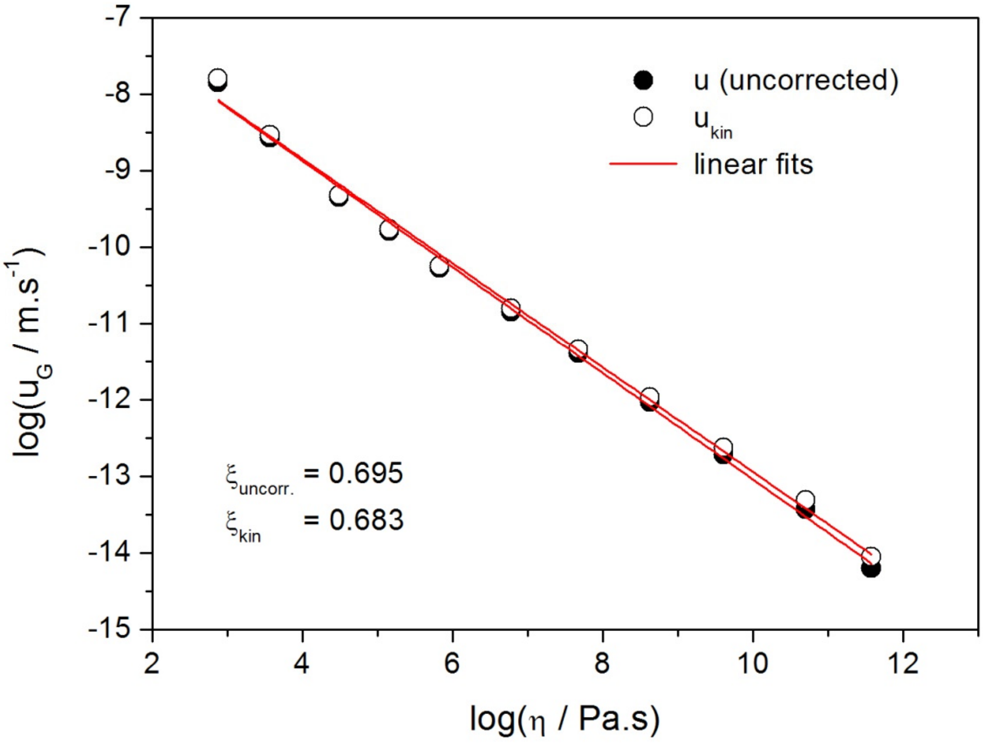

Figure S3: Evaluation of the decoupling parameter ξ.
